# Steerable esophageal thermometer for atrial fibrillation ablation in a patient with esophageal achalasia: a case report

**DOI:** 10.1002/ccr3.1439

**Published:** 2018-03-10

**Authors:** Hidehira Fukaya, Shinichi Niwano, Sho Ogiso, Yuki Arakawa, Ai Horiguchi, Ryo Nishinarita, Hironori Nakamura, Jun Oikawa, Akira Satoh, Jun Kishihara, Junya Ako

**Affiliations:** ^1^ Department of Cardiovascular Medicine Kitasato University School of Medicine Sagamihara Japan

**Keywords:** Atrial fibrillation, complication, esophagus

## Abstract

Esophageal injury is a major concern during catheter ablation of atrial fibrillation. Operators avoid radiofrequency applications on the esophagus by changing ablation line; however, it is unavoidable in patients with a dilated esophagus, such as esophageal achalasia. Steerable esophageal thermometer is useful for evaluating precise temperatures to prevent esophageal injury.

## Introduction

A 72‐year‐old man was referred to our hospital for treatment of brady‐tachycardia syndrome with paroxysmal atrial fibrillation. He had esophageal achalasia. Computed tomography revealed that the entire posterior wall of left atrium directly contacting dilated esophagus. Radiofrequency catheter ablation was performed with precise temperature monitoring by steerable esophageal thermometer.

Esophageal injury during catheter ablation of atrial fibrillation (AF) sometimes becomes fatal 1, 2. To avoid any esophageal injury, temperature monitoring with an esophageal thermometer is useful in catheter ablation 3. However, as the esophageal diameter is about 2 cm, a thin catheter would not be able to cover the entire esophageal area, which might give the operator a false feeling of safety when ablating on the posterior wall adjacent to the esophagus. Here, we report an AF patient with esophageal achalasia undergoing radiofrequency catheter ablation of AF with the use of a steerable esophageal thermometer.

## Case

**Figure 1:**
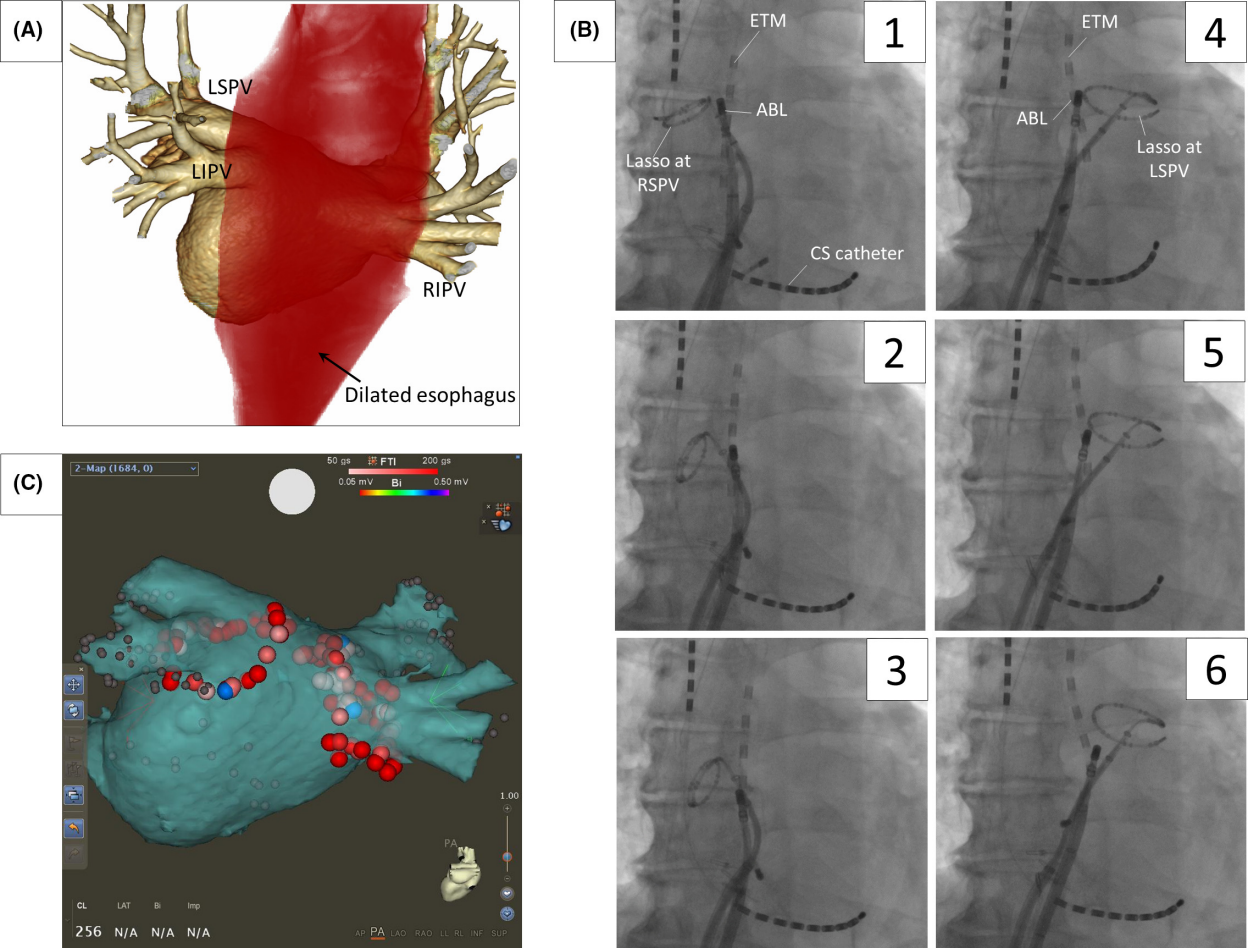
(A) Three‐dimensional computed tomography revealed a dilated esophagus facing the entire posterior wall of the LA. The red shadow indicates the dilated esophagus. (B) The Esophastar^®^ was tentatively placed at the right (1–3) and left (4–6) posterior ablation sites. (C) The EEPVI was successfully performed using CARTO3. The tags indicate the ablation points for the EEPVI. LSPV, left superior pulmonary vein; LIPV, left inferior pulmonary vein; RIPV, right inferior pulmonary vein; ETM, esophageal temperature monitor; ABL, ablation catheter; CS, coronary sinus; EEPVI, extensive encircling pulmonary vein isolation.

A 72‐year‐old male was referred to our hospital for treatment of brady‐tachycardia syndrome (BTS). He had esophageal achalasia and underwent per‐oral endoscopic myotomy (POEM) 2 weeks prior. During his hospital stay, he was diagnosed with repeated episodes of paroxysmal AF followed by sinus arrest with presyncopal symptoms, that is, BTS. After the POEM, the esophagus was still dilated and computed tomography revealed that the dilated esophagus came in contact with the entire posterior wall of the left atrium (Figure 1A). Concerned about esophageal injury, it was decided to perform radiofrequency catheter ablation of AF using a steerable esophageal thermometer to precisely assess the esophageal temperature.

Radiofrequency catheter ablation (RFCA) was performed under general anesthesia with mechanical ventilation using a laryngeal mask. At first, to reduce the esophageal inner pressure, a gastric tube was inserted and properly drained. Then, a steerable esophageal thermometer (Esophastar^®^, Japan Lifeline Co., Ltd, Japan) was inserted. An extensive encircling pulmonary vein isolation (EEPVI) was performed with an irrigation catheter (thermoCool STSF^®^, Biosense Webster, Diamond Bar, CA) using CARTO3^®^ (Biosense Webster). Standard anterior ablation lines were applied with 30 W of energy and 5–20 g of contact pressure based on a force–time integral (FTI) target of over 200 gs. When performing the linear ablation on the posterior wall, the esophageal thermometer was positioned just on the opposite site of the ablation catheter in the esophagus (Figure 1B1–6). Ablation was interrupted when the esophageal temperature exceeded 40°C even if the FTI did not reach 200 gs. The EEPVI was successfully achieved (Figure 1C) without any dormant conduction induced by a 2 *μ*g isoproterenol and 20 mg adenosine triphosphate injection. No non‐PV foci were induced by an isoproterenol infusion nor burst pacing.

Esophageal endoscopy performed on the next day revealed no abnormal findings of the mucosa of the entire esophagus. After the RFCA of AF, no sinus arrest was observed. He has been free from any symptoms for 3 months after the procedure without any antiarrhythmic drugs.

## Discussion

The prevalence of esophageal injury during AF ablation is reported to be approximately 30–47% 2, 4, including asymptomatic slight erythemas or erosions of the mucosa 5, 6, 7. They do not usually become severe complications with the use of antacids such as proton pump inhibitors 8. However, atrioesophageal fistulae, although rare (0.02–0.4%) 7, 9, 10, are devastating and have a high mortality rate of up to 70% 1, 2, 7. The positional relationship between the esophagus and LA, individually, varies and may sometimes change during the procedure even in the same patient 11. Esophageal thermometers are useful to prevent any esophageal injury during AF ablation 3. However, as the diameter of these catheters is ≤4 mm, they of course cannot fully cover the entire esophagus. Although a unique S‐curved esophageal thermometer has been launched to complement this issue, it does not fit a dilated esophagus as observed in this patient. Therefore, we used a steerable esophageal thermometer (Esophastar^®^) to position it in relation to the ablation sites in order to evaluate more accurate temperatures.

Muller et al. reported that the use of esophageal temperature probes was paradoxically related to a higher incidence of esophageal injury 6. They mentioned that a possible mechanism of this result might be an “antenna effect” of the metallic tip of the catheter; that is, direct thermal injury from the temperature probe, whereas this remains controversial 12, 13. We set the upper limit of the esophageal temperature at 40°C during the procedure as a safety margin because it has been reported that setting it at less than 40–41°C was associated with a lower complication rate of esophageal injury 5, 7, 14; however, there is no clear cutoff value for the temperature setting to avoid esophageal thermal injury 8, 15. As a result, this case achieved a successful AF ablation without any esophageal complications using the Esophastar^®^. The Esophastar^®^ has uncoated thermocouples, which may have an antenna effect as mentioned above; however, we could avoid an excessive radiofrequency inductive heating of the probes by setting an upper limit of the esophageal temperature. Additionally, another possible mechanism might be “false safety information”, which can be provided by the esophageal thermometer when it was anatomically some distance from the real ablation site even in cases with a regular‐sized esophagus. This false information may paradoxically lead to an inappropriately high‐energy delivery. To avoid this kind of pitfall, we delivered the radiofrequency energy by placing the esophageal thermometer just on an opposite site of the ablation site with a temperature setting of less than 40°C. The relationship between an increase in the esophageal temperature and esophageal injury has to an extent already been established 3, 5, 7, 16. Although the mechanisms of a paradoxical increase in esophageal injury with esophageal thermometer remain unclear, this methodology may also have helped provide a high safety level in this case. As there is no clear evidence about the safety for evaluating real‐time esophageal temperatures during RFCA using an esophageal thermometer, randomized control studies are necessary to conclude this issue.

## Conclusion

Steerable esophageal thermometer is useful when performing RFCA of AF to prevent any esophageal injury in patients with a dilated esophagus due to esophageal achalasia.

## Conflict of Interest

None.

## Authorship

HF: wrote the manuscript. SN: revised the manuscript as the senior author. SO: corrected the clinical data and reviewed the manuscript. YA, AH, RN, HN, JO, AS, and JK: performed the catheter ablation with the corresponding author and revised the manuscript. JA: reviewed and revised the final manuscript as the senior author.

## References

[1] Schuring, C. A. , L. J. Mountjoy , A. B. Priaulx , R. J. Schneider , H. L. Smith , G. C. Wall , et al. 2017 Atrio‐esophageal fistula: a case series and literature review. Am. J. Case Rep. 18:847–854. 28761039 10.12659/AJCR.903966 PMC5551930

[2] Cappato, R. , H. Calkins , S. A. Chen , W. Davies , Y. Iesaka , J. Kalman , et al. 2009 Prevalence and causes of fatal outcome in catheter ablation of atrial fibrillation. J. Am. Coll. Cardiol. 53:1798–1803. 19422987 10.1016/j.jacc.2009.02.022

[3] Singh, S. M. , A. d'Avila , S. K. Doshi , W. R. Brugge , R. A. Bedford , T. Mela , et al. 2008 Esophageal injury and temperature monitoring during atrial fibrillation ablation. Circ. Arrhythm. Electrophysiol. 1:162–168. 19808410 10.1161/CIRCEP.107.789552

[4] Schmidt, M. , G. Nolker , H. Marschang , K. J. Gutleben , V. Schibgilla , H. Rittger , et al. 2008 Incidence of oesophageal wall injury post‐pulmonary vein antrum isolation for treatment of patients with atrial fibrillation. Europace 10:205–209. 18256125 10.1093/europace/eun001

[5] Halm, U. , T. Gaspar , M. Zachaus , S. Sack , A. Arya , C. Piorkowski , et al. 2010 Thermal esophageal lesions after radiofrequency catheter ablation of left atrial arrhythmias. Am. J. Gastroenterol. 105:551–556. 19888201 10.1038/ajg.2009.625

[6] Muller, P. , J. W. Dietrich , P. Halbfass , A. Abouarab , F. Fochler , A. Szollosi , et al. 2015 Higher incidence of esophageal lesions after ablation of atrial fibrillation related to the use of esophageal temperature probes. Heart Rhythm 12:1464–1469. 25847474 10.1016/j.hrthm.2015.04.005

[7] Halbfass, P. , B. Pavlov , P. Muller , K. Nentwich , K. Sonne , S. Barth , et al. 2017 Progression from esophageal thermal asymptomatic lesion to perforation complicating atrial fibrillation ablation: a single‐center registry. Circ. Arrhythm. Electrophysiol. 10:e00523. 10.1161/CIRCEP.117.005233 28798021

[8] Di Biase, L. , M. Dodig , W. Saliba , A. Siu , J. Santisi , S. Poe , et al. 2010 Capsule endoscopy in examination of esophagus for lesions after radiofrequency catheter ablation: a potential tool to select patients with increased risk of complications. J. Cardiovasc. Electrophysiol. 21:839–844. 20163496 10.1111/j.1540-8167.2010.01732.x

[9] Dagres, N. , G. Hindricks , H. Kottkamp , P. Sommer , T. Gaspar , K. Bode , et al. 2009 Complications of atrial fibrillation ablation in a high‐volume center in 1,000 procedures: still cause for concern? J. Cardiovasc. Electrophysiol. 20:1014–1019. 19490383 10.1111/j.1540-8167.2009.01493.x

[10] Cappato, R. , H. Calkins , S. A. Chen , W. Davies , Y. Iesaka , J. Kalman , et al. 2010 Updated worldwide survey on the methods, efficacy, and safety of catheter ablation for human atrial fibrillation. Circ. Arrhythm. Electrophysiol. 3:32–38. 19995881 10.1161/CIRCEP.109.859116

[11] Good, E. , H. Oral , K. Lemola , J. Han , K. Tamirisa , P. Igic , et al. 2005 Movement of the esophagus during left atrial catheter ablation for atrial fibrillation. J. Am. Coll. Cardiol. 46:2107–2110. 16325049 10.1016/j.jacc.2005.08.042

[12] Perez, J. J. , A. D'Avila , A. Aryana , and E. Berjano . 2015 Electrical and thermal effects of esophageal temperature probes on radiofrequency catheter ablation of atrial fibrillation: results from a computational modeling study. J. Cardiovasc. Electrophysiol. 26:556–564. 25648533 10.1111/jce.12630

[13] Back Sternick, E. , A. Cohen Persiano , and V. Arantes . 2012 Is it safe to monitor oesophageal temperature during AF ablation? Europace 14:1432. 22613665 10.1093/europace/eus065

[14] Sause, A. , O. Tutdibi , K. Pomsel , W. Dinh , R. Futh , M. Lankisch , et al. 2010 Limiting esophageal temperature in radiofrequency ablation of left atrial tachyarrhythmias results in low incidence of thermal esophageal lesions. BMC Cardiovasc. Disord. 10:52. 20977747 10.1186/1471-2261-10-52 PMC2987899

[15] Nakagawa, H. , K. A. Seres , and W. M. Jackman . 2008 Limitations of esophageal temperature‐monitoring to prevent esophageal injury during atrial fibrillation ablation. Circ. Arrhythm. Electrophysiol. 1:150–152. 19808408 10.1161/CIRCEP.108.805366

[16] Musat, D. , and S. Mittal . 2011 The esophageal temperature probe: helpful monitoring device or inadvertent amplifier of risk? J. Cardiovasc. Electrophysiol. 22:262–264. 20958839 10.1111/j.1540-8167.2010.01936.x

